# Psychological Behavior of Frontline Medical Staff in the Use of Preventive Medication for COVID-19: A Cross-Sectional Study

**DOI:** 10.3389/fpsyg.2020.02104

**Published:** 2020-09-25

**Authors:** Xiaoyan Yu, Yuxi Li, Li Tang, Lu Deng, Yuxin Zhao, Xianmei Zhao, Huilan Xu, Ming Zeng

**Affiliations:** ^1^Department of Social Medicine and Health Management, Xiangya School of Public Health, Central South University, Changsha, China; ^2^Department of Clinical Nursing, The Second Xiangya Hospital of Central South University, Changsha, China; ^3^Department of Health Toxicology, Xiangya School of Public Health, Central South University, Changsha, China; ^4^Department of Health Management Center, The Second Xiangya Hospital of Central South University, Changsha, China

**Keywords:** COVID-19, preventive medication, combined Chinese and Western medicine treatment, psychological, medical staff in fighting against COVID-19

## Abstract

**Purpose:**

To understand the current pandemic, levels of anxiety in frontline staff, and whether they have been using medication to prevent COVID-19.

**Methods:**

Between January 10 and March 10, 2020, 290 frontline staff completed a questionnaire incorporating the Generalized Anxiety Disorder Scale 7 (GAD-7) to indicate their psychological behavior in the use of preventive medication.

**Results:**

Of those who participated in the study, 77.6% used preventive medication, with 47.5, 40.9, and 11.6% using these as part of routine preventive treatment, to fight infection after it was contracted, and after occupational exposure, respectively. There was a statistically significant relationship between the anxiety scale scores and the frequency of medication use (*P* < 0.05). Comparative analyses revealed that the scores of those in the group taking medication after occupational exposure (to respiratory and blood-borne pathogens) were significantly different from other groups. The proportion of participants choosing Western medicine, traditional Chinese medicine, and integrated Chinese and Western medicine was 24.4, 28.0, and 47.6%, respectively. Additionally, the relationship between the anxiety scale scores and the three types of medication was statistically significant (*P* < 0.05), as was the difference between Western medicine and other groups. According to Multinomial logistic regression based on the adjustment to gender, age, educational level, marital status, current workplace, and profession, participants with moderate to severe anxiety, had higher odds (*OR* = 10.331, 95%CI:1.453–73.429) of using Western medicine than participants with no anxiety. Participants with moderate anxiety were 6.399 times more likely to use an integrated combination of traditional Chinese and Western medicine compared to those with no anxiety (*OR* = 6.399, 95%CI:1.007–40.658). Furthermore, those with mild anxiety were 2.656 times more likely to use integrated traditional Chinese and Western medicine than those with no anxiety (*OR* = 2.657, 95%CI:1.075–6.570). The probability that frontline medical staff with moderate anxiety took preventive medication after occupational exposure to COVID-19 was 8.066 times (*OR* = 8.066, 95%CI:1.043–62.353) higher than that of staff without anxiety.

**Discussion:**

This study revealed that there was more anxiety among frontline medical staff who took medication after unexpected occupational exposure. There was less anxiety among those using an integrated course of Chinese and Western medicine than Western medicine alone. It was also observed that anxiety affects the types and frequency of the preventive medication taken. Frontline medical staff who suffer from anxiety are also more likely to use medication to prevent COVID-19.

## Introduction

Coronavirus disease (COVID-19) is caused by severe acute respiratory syndrome coronavirus 2 (SARS-CoV-2). It was first identified in December 2019 in Wuhan, China. A worldwide pandemic ensued, and a global state of emergency has been declared, with over 200,000 COVID-19 cases confirmed in 166 countries and regions by March 18, 2020 (World Health Organization, 2020a). Like many other countries worldwide, China has undertaken concerted efforts to develop medical treatments, scientific research, public health responses, and other methods for tackling the prevention and control of infection as a matter of urgency, and frontline medical staff are the core force in progressing the treatment of patients with COVID-19.

At present, there are no antiviral drugs or vaccines, or preventive medicine specific to COVID-19: treatment consists of symptomatic therapy only. The preventive measures usually implemented for SARS-CoV-2 include strict disinfection and isolation procedures, enhanced occupational exposure risk management, and enhanced immunity, however, these cannot offer frontline staff the required protection under these increased pressures and taking into account infection risk, and stress in their current working environment. Consequently, it is vital to ensure the safety of these staff and prevent their infection by COVID-19 (World Health Organization, 2020b). In the absence of clear medical guidelines, some frontline medical staff are turning to medications to prevent or control the risk of contracting COVID-19, or when displaying respiratory symptoms. The correlation between anxiety and the use of preventive medication among frontline medical staff, and how it influences their efforts to stay healthy, is still unknown (Kang et al., 2020). Therefore, this study investigates the current situation of COVID-19 prevention and provides a theoretical basis for more specific pandemic prevention and control measures.

## Methods

### Study Design and Data Collection

Between January 10 and March 10, 2020, a cross-sectional survey was conducted in three Chinese COVID-19-designated hospitals. Through simple random sampling, 290 frontline clinical, medical, and public health staff from Hunan, Guangdong, and Hubei Provinces agreed to participate. In this study, frontline medical staff are defined as those in contact with new confirmed or suspected COVID-19 cases or samples. The researchers first introduced the purpose and significance of the survey after obtaining their consent and guided the participants to complete the specially designed online questionnaire, which took 3–5 min. All 290 questionnaires were submitted, a rate of 100% completion and effective recovery.

### Demographic Variables and Work Characteristics

The self-reported attributes collected for each participant included: gender; age, grouped as <30, 30–40, or >40; educational level, ranked as Up to junior college, College, or Graduate or higher; marital status, categorized as Unmarried, Married, or Other; and profession, identified as Clinician, Nurse, Laboratory Technician, and Public Health Worker.

### Use of Preventive Medication Among Frontline Medical Staff

The use of preventive medication was assessed through seven items that were specially designed following a literature review and pilot survey of frontline medical staff. The first three items requested: the name of any medication used; when they were used; and how often they were used to prevent contracting COVID-19. The frequency of use could be indicated by either: In accordance with the instructions; Used when an infection suspected; or Used after occupational exposure. The final four items investigated the attitude of frontline medical staff toward preventive medication by measuring: the level of risk from COVID-19 at which they considered themselves to be; the extent of their concern over their health; and the effectiveness they believed the medication to have in preventing infection. These were measured as Low; Medium; High; Very High; and whether they were worried about the side effects of the medication.

### Anxiety Levels Among Frontline Medical Staff

Anxiety levels among frontline medical staff were measured using Spitzer et al.’s (2006) Generalized Anxiety Disorder Scale 7 (GAD-7) since it is widely used for screening clinical anxiety and considered reliable due to its Cronbach α coefficient of 0.898. Reflecting on their feelings over the previous 2 weeks, respondents reported their degree of fear for seven items: Not at all (0); Several days (1); More than half of the days (2); or Almost every day (3). The values of each item were totaled to produce an overall score ranging from 0 to 21, indicating anxiety levels as follows: No anxiety (0–4); Mild anxiety (5–9); Moderate anxiety (10–13); Moderate to severe anxiety (14–18); and Severe anxiety (19–21).

### Statistical Methods

All the statistical analyses were performed using IBM^®^ SPSS^®^ Statistics 23.0, and a two-tailed probability value of <0.05 was considered statistically significant. The current situation of preventive medication of frontline medical staff was statistically described. A one-way analysis of variance (ANOVA) was undertaken to compare the differences in scores for each of the scale item groups, including those for the frequency of medication use and types of medicine. *Post-hoc* tests were performed, comparing anxiety levels under different circumstances: LSD test was used in homogeneity variances, and Tamhane’s T2 test was used in non-homogeneity variances. Multinomial logistic regression was undertaken to explore the anxiety levels affecting the use of preventive medication under the control of socio-demographic variables.

## Results

### General Attributes of Frontline Medical Staff

The average age of the 186 female and 104 male respondents, aged between 22 and 52, is 31 years old. As shown in Table 1, the majority have completed a college or higher education (90.7%) and worked as clinicians or nurses (85.9%), while over two-thirds of the respondents work in Hunan Province (70.7%).

**TABLE 1:** General attributes of frontline medical staff.

Variable	Group	N	Proportion (%)
Gender	Male	104	35.9
	Female	186	64.1
Age	<30	126	43.4
	30–40	140	48.3
	>40	24	8.3
Educational level	Up to junior college or lower	27	9.3
	College	195	67.2
	Graduate or higher	68	23.5
Marital status	Unmarried	116	40
	Married	169	58.3
	Other	5	1.7
Current workplace	Hunan Province	205	70.7
	Hubei Province	15	5.2
	Guangdong Province	70	24.1
Profession	Clinician	76	26.2
	Nurse	173	59.7
	Laboratory technician	12	4.1
	Public health worker	29	10

### Current Use of Preventive Medication Among Frontline Medical Staff

**FIGURE 1:**
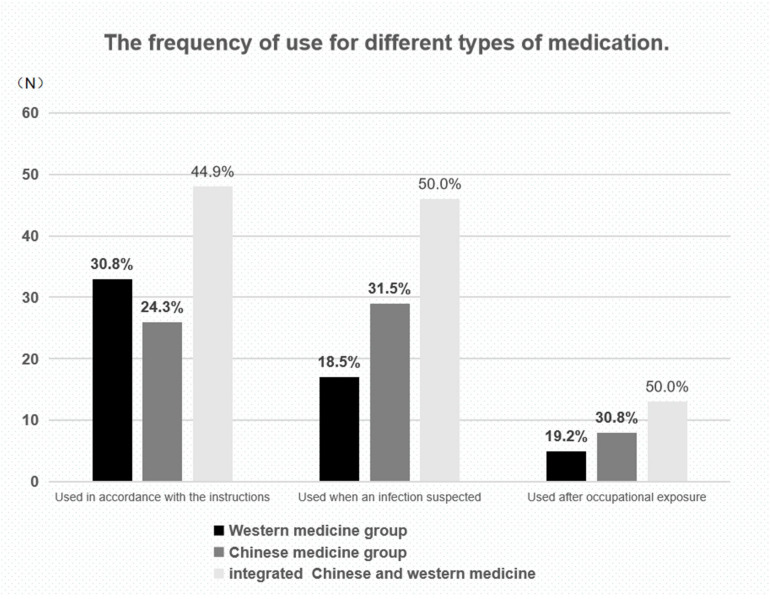
Types of medication and frequency of use.

Table 2 shows the types and names of preventive medications and Figure 1 shows the medication used and reveals that the majority of participants used integrated Chinese and Western medicine. However, the second most-used medication is traditional Chinese medicine among frontline medical staff that have either displayed respiratory symptoms or been exposed to the virus while working, Western medicine is used among those choosing to take preventive medication.

**TABLE 2:** Types and names of preventive medications used by frontline medical staff.

Type	Drug function and name
Western medicine	Immunomodulator: thymalfasin, pidotimod, immunoglobulins, Siqikang
	Antivirals:oseltamivir, arbidol
Traditional Chinese medicine	Antivirals: radix isatidis, bupleurum, Lianhua Qingwen capsules, Chinese medicine prescription
Integrated Chinese and western medicine	Immunomodulator with traditional Chinese antiviral (and/or) western antiviral

### Comparison of Anxiety Levels Among Medical Staff Under Different Circumstances

The scores from the GAD-7 were assigned as follows: 0 scores for No anxiety symptoms; 1 score for Low anxiety; 2 scores for Moderate anxiety; 3 scores for Moderate to severe anxiety; and 4 scores for Severe anxiety. The results of the one-way ANOVA for the use of preventive medication compared to anxiety levels among frontline staff are shown in Table 3.

**TABLE 3:** Comparison of anxiety levels under different circumstances.

Category	Group	N	Anxiety score ($\bar{x}$ ± s)	*F*	*P*
	No medicine	65	0.75 ± 0.94^a^		
Frequency of medicine use	Used in accordance with the instructions	107	0.87 ± 0.93^a^	2.792	0.041
	Used when an infection suspected	92	0.75 ± 0.79^a^		
	Used after occupational exposure	26	1.31 ± 1.16		
	No medicine	65	0.75 ± 0.94^b^		
Type of medicine	Western medicine group	55	1.29 ± 1.18	5.984	0.001
	Chinese medicine group	63	0.63 ± 0.90^b^		
	Integrated Chinese and western medicine	107	0.79 ± 0.68^b^		

*Following a further least significance difference (LSD) test. ^*a*^Indicates the comparison with the drug used after occupational exposure (P < 0.05). ^*b*^Indicates the comparison with Western medicine (P < 0.05).*

The anxiety level scores for the different frequencies of use indicate a statistically significant difference (*P* = 0.041) in groups. A comparison of the two groups by LSD showed that the anxiety level scores for use after occupational exposure are higher than with no medicine group (*P* = 0.010), which is in accordance with results from the instruction group (*P* = 0.029), and use when an infection suspected group (*P* = 0.006). The anxiety level scores for use after occupational exposure are higher than in the other groups. Similarly, the anxiety level scores for the different types of medication are significantly different statistically (*P* = 0.002), with those in the Western medicine group being higher. Comparisons between every two groups by Tamhane’s T2 showed that the anxiety level scores for use of Western medicine groups are higher than the no medicine group (*P* = 0.045), Chinese medicine group (*P* = 0.007), and Integrated Chinese and Western medicine group (*P* = 0.031). Furthermore, Compared with Western medicine, the use of Integrated Chinese and Western medicine may relieve anxiety among frontline medical staff who work directly with COVID-19 patients and samples.

### Analysis of Anxiety Levels and Preventive Medication in Adjusted Socio-Demographic Variables

In Table 4, a Multinomial logistic regression was established by taking the types of medicine as the dependent variable, the anxiety levels, and other factors that may affect the results (gender, age, education level, marital status, work location, and occupation) function as the independent variables. Participants with moderate to severe anxiety were merged because of relatively limited cases in each severe anxiety group. Those with moderate to severe anxiety had higher odds (*OR* = 10.331, 95%CI:1.453–73.429) of using Western medicine than participants with no anxiety. Participants with moderate anxiety were 6.399 times more likely to use integrated traditional Chinese and Western medicine, compared to those with no anxiety (*OR* = 6.399, 95%CI:1.007–40.658). Furthermore, those with mild anxiety had the probability of 2.656 that they were likely to use integrated traditional Chinese and Western medicine than those with no anxiety (*OR* = 2.657, 95%CI:1.075–6.570).

**TABLE 4:** Relationship between the medication and anxiety in Multinomial logistic regression.

Dependent variable^†^	Anxiety levels OR (95%CI)		
	Mild vs. None	Moderate vs. None	Moderate to severe^#^ vs. None
**Type of medication**			
No medicine (reference)	–	–	–
Western medicine group	1.596 (0.562–4.534)	4.836 (0710–32.936)	10.331 (1.453–73.429)*
Chinese medicine group	0.724 (0.290–1.807)	1.283 (0.182–9.025)	2.121 (0.299–15.048)
Integrated Chinese and western medicine	2.657 (1.075–6.570)*	6.399 (1.007–40.658)*	1.282 (0.121–13.650)
**Frequency of medication**			
No medicine (reference)	–	–	–
Used in accordance with the instructions	1.432 (0.608–3.371)	2.301 (0.403–13.147)	5.394 (0.835–34.839)
Used when an infection suspected	1.196 (0.505–2.834)	2.789 (0.501–15.517)	0.862 (0.093–7.969)
Used after occupational exposure	2.302 (0.639–8.298)	8.066 (1.043–62.353)*	7.993 (0.821–77.779)

^#^The cases of moderate to severe group and severe group were merged in Multinomial logistic regression. ^†^Adjusted for gender, age, educational level, marital status, current workplace, and profession. *Statistically significant at α = 0.05.

Similarly, a Multinomial logistic regression was applied to explore the influence that the anxiety levels may have on the frequency of medication use. It has been shown that the probability that frontline medical staff with moderate anxiety took preventive medication after occupational exposure was 8.066 (*OR* = 8.066, 95%CI:1.043–62.353) times more than that of staff without anxiety.

## Discussion

### Current Use of Preventive Medication by Frontline Medical Staff

This study investigated the current use of preventive medication against COVID-19 among frontline medical staff facing different risks. Of the 290 participants from Hunan, Guangdong, and Hubei Provinces, 225 took preventive medication, with 47.5% taking medication according to the instructions, 40.9% when they were facing a suspected infection, and 11.6% after occupational exposure. The medications listed by the frontline medical staff included bot intravenous (immunomodulators) and oral drugs (antivirals). The main reason for using preventive medication was the fact that no vaccine is currently available for COVID-19 (Lu, 2020), meaning that even when staff wore personal protective equipment (PPE), the threat of infection remains.

Both doctors and nurses come into close contact with COVID-19 patients, for example when taking sputum samples, establishing artificial airways, and performing bronchoscopy (World Health Organization, 2020b). Public health workers are also helpful for screening procedures and supporting those in isolation, such as undertaking epidemiological investigations, disinfecting contaminated areas, and performing nucleic acid detection tests. Thus, because they are in direct contact with confirmed and suspected cases of COVID-19 and samples, frontline medical staff are more likely to be exposed, are at high risk of infection in the workplace, and subject to the medical observation period.

In the absence of specific drugs and vaccines, the guidelines for Chinese Novel Coronavirus Pneumonia do recommend some traditional Chinese medicines for those in the medical observation period (National Health Commission, 2020). This study found that not only traditional Chinese medicine (e.g., Lianhua Qingwen capsules, Chinese medicine prescriptions) but also Western medicine (e.g., Thymalfasin, Arbidol) were used. Of the 225 participants who took preventive medication, 24.4% chose Western medicine, 28.0% traditional Chinese medicine, and 47.6% integrated Chinese and Western medicine. Moreover, 72.4% of the drugs were prescribed by doctors and dispensed by the hospitals in which they worked. Scholars have simultaneously suggested the rational use of drugs and close observation for any reactions (Jiao et al., 2020). Indeed, the frontline medical staff in this study reported some adverse reactions, including diarrhea, nausea, and dizziness, with 50.4% worried it would affect their fitness to work. Of these, 35.8% had high confidence that preventive medications can help the body fight COVID-19.

### Anxiety Levels Among Frontline Medical Staff Taking Medications After Occupational Exposure

The findings of this study showed that 85% of frontline medical staff were highly concerned about their health and 41.1% thought that their risk of COVID-19 infection was high. Sixty percentage of frontline medical staff experienced anxiety and other negative emotions, such as fear and worry. Among the 225 using preventive medications, 11.6% reported taking immunomodulators and antivirals after unexpected occupational exposure. This occurred following accidental incidents, such as damage to PPE or a needle-stick injury, leading to direct contact with or inhalation of droplets and secretions containing pathogens. Once this happens, staff are required to temporarily stop work and enter the 14-day medical observation period. At present, there is no consensus on a contingency plan among medical institutions, with each formulating schemes according to specific situations: providing emergency treatment to wounds, spraying exposed parts with alcohol, leaving the contaminated area, reporting the incident, and giving preventive medication after exposure. The traumatic experience and acute stress caused by these incidents may explain the higher anxiety levels among those in this study who used medication after occupational exposure. This tendency reflects the fact that most frontline medical staff are encountering a serious epidemic situation in which they suffer considerable stress in a short period of time (National Health Commission of the People’s Republic of China, 2020).

### Anxiety Levels Among Frontline Medical Staff Taking Integrated Chinese and Western Medicine

Historically, the use of traditional Chinese medicine to treat infections is based on principles such as strengthening the body’s resistance to eliminate pathogenic factors, and syndrome differentiation and treatment. Thus, it often involves improving immunity and individual symptomatic treatment. To date, China has achieved good results by combining traditional Chinese with Western medicine (Luo et al., 2020; Ni et al., 2020; Wang et al., 2020): using traditional Chinese medicine and strict isolation procedures has greatly helped with the prevention and control of COVID-19 for frontline medical staff and others who have had close contact with the virus (Ling, 2020). In addition, the *COVID-19 Prevention and Treatment Program* in Hubei Province recommends integrated Chinese and Western medicine to treat COVID-19. Chinese medicine prescriptions for those at high risk are: astragalus, 15 g; fried atractylodes, 9 g; wind, 9 g; cyrtomium rhizome, 6 g; jin yin hua, 9 g; dried tangerine or orange peel, 6; and perrin, 9 g (Ba et al., 2020). Both traditional Chinese and Western medicines are uniquely beneficial in the treatment of diseases, but the effectiveness of their combined use requires further research. Nevertheless, anxiety levels among those who chose both in this study were lower than those using only Western medicine; however, this may be due to the effectiveness of the individual’s belief in its benefits. Moreover, no cases of COVID-19 have been reported among this group of frontline medical staff to date.

### Influences on the Use of Preventive Medication Caused by Anxiety

According to the Multinomial logistic regression based on the adjustment to gender, age, educational level, marital status, current workplace, and profession, when suffering from anxiety symptoms, frontline medical staff tended to use medication to prevent the COVID-19. Furthermore, with occupational exposure and moderate anxiety symptoms, they were more likely to use preventive medication to prevent COVID-19 than those with no anxiety. Anxiety symptoms were a risk factor in the use of preventive medication among the frontline medical staff. This is possibly due to worries about the high-risk of COVID-19 infection in the designated hospitals. For some frontline medical staff who took part in fighting against COVID-19, this was the first time that faced a serious public health emergency, which posed several challenges on their professional skills, occupational protection, and psychological tolerance (Lai et al., 2020). Thus, their body and mind are full of stress (Trotman et al., 2018). Faced with working stress, they were suffering from fatigue, headache, insomnia, sweating, palpitations, and other physical symptoms. As a result, they reported self-doubt about the symptoms of COVID-19, and this could have led to their increased use of preventive medication. When the frontline medical staff reported self-doubt about the symptoms of COVID-19, physicians tended to prescribe the use of Western medicines or integrated Chinese and Western medicine as pre-exposure medications.

This prediction model reminds us that more attention should be paid to the frontline medical staff with occupational exposure. If staff met with occupational exposure, they are more likely to have symptoms of serve anxiety and tend to use preventive medication.

This discovery reminds hospital managers that the relevant functional department can relieve frontline medical staff from their anxiety and stress in the following aspects: an increase in environmental safety, rational team management, and professional psychological intervention (Tang et al., 2020). Meanwhile, it is suggested that a contingency plan for occupational exposure should be established and preventive medication should be more standardized in medical institutions.

### Limitation

This study is limited because of the size of the sample population and the inclusion of only three of China’s major cities. Therefore, the conclusions, reliability, and generalizations of the study are yet to be tested. Further research is required to verify the effectiveness of integrated Chinese and Western medicine in the prevention and treatment of COVID-19.

## Conclusion

This study revealed more anxiety among frontline medical staff taking medication after unexpected occupational exposure and less among those using integrated Chinese and Western medicine than Western medicine alone. When suffering the symptoms of anxiety, frontline medical staff tend to use medication to prevent COVID-19. Furthermore, when they had symptoms of occupational exposure and moderate anxiety, they were more likely to use preventive medication to prevent COVID-19 than those with no anxiety. These further problems related to preventing the COVID-19 epidemic still need to be addressed and it is important to support and maintaining the physical and mental health of frontline medical staff, whilst also reducing the likelihood of frontline staff contracting the virus.

## Data Availability Statement

The raw data supporting the conclusions of this article will be made available by the authors, without undue reservation, to any qualified researcher.

## Ethics Statement

The studies involving human participants were reviewed and approved by the Ethics Committee of Xiangya School of Public Health of Central South University. The patients/participants provided their written informed consent to participate in this study. Written informed consent was obtained from the individual(s) for the publication of any potentially identifiable images or data included in this article.

## Author Contributions

XY, YL, HX, XZ, and MZ contributed to the study design and data collection. YL conducted the data analysis. XY, LT, and YL drafted the manuscript. YZ and LD revised the manuscript. All authors contributed to editing the manuscript and approved the final version for publication.

## Conflict of Interest

The authors declare that the research was conducted in the absence of any commercial or financial relationships that could be construed as a potential conflict of interest.

## Abbreviations

COVID-19: Coronavirus disease 2019
GAD-7: Generalized Anxiety Disorder Scale 7.
